# CircRNAs: role in human diseases and potential use as biomarkers

**DOI:** 10.1038/s41419-021-03743-3

**Published:** 2021-05-11

**Authors:** Lorena Verduci, Emilio Tarcitano, Sabrina Strano, Yosef Yarden, Giovanni Blandino

**Affiliations:** 1 Unit of Oncogenomic and Epigenetic, Department of Research, Advanced Diagnostic, and Technological Innovation, IRCCS, Regina Elena National Cancer Institute, Rome, Italy; 2 Department of Biological Regulation, Weizmann Institute of Science, Rehovot, Israel; 3 SAFU Unit, Department of Research, Advanced Diagnostic, and Technological Innovation, IRCCS, Regina Elena National Cancer Institute, Rome, Italy

**Keywords:** Cancer, Cell biology, Molecular biology

## Abstract

Circular RNAs (circRNAs) are a class of endogenous RNAs characterized by a covalent loop structure. In comparison to other types of RNAs, the abundance of circRNAs is relatively low but due to the circular configuration, their stability is very high. In addition, circRNAs display high degree of tissue specificity. The sponging activity of circRNAs toward microRNAs is the best-described mode of action of circRNAs. However, the ability of circRNAs to bind with specific proteins, as well as to encode short proteins, propose alternative functions. This review introduces the biogenesis of circRNAs and summarizes the roles played by circRNAs in human diseases. These include examples of their functional roles in several organ-specific cancers, such as head and neck and breast and lung cancers. In addition, we review potential functions of circRNAs in diabetes, cardiovascular, and neurodegenerative diseases. Recently, a growing number of studies have demonstrated involvement of circRNAs in a wide spectrum of signaling molecular pathways, but at the same time many different and controversial views on circRNAs role and function are emerging. We conclude by offering cellular homeostasis generated by networks comprising circular RNAs, other non-coding RNAs and RNA-binding proteins. Accordingly, it is predictable that circRNAs, due to their highly stable nature and remarkable tissue specificity, will emerge as reliable biomarkers of disease course and treatment efficacy.

## Facts

- circRNAs are single-stranded circles of RNA, which form highly stable closed loops.
- circRNAs can have different functions. Among these, the miRNA sponging is the best-characterized role.
- circRNAs are widely expressed in human tissues and their expression is highly tissue-specific.
- circRNAs are involved in many human diseases, including cancer and neurodegenerative disorders.
- The biochemical characteristics of circRNAs, especially stability and tissue specificity, make them ideal biomarkers for clinical use.

## Open questions

- Are there yet unknown features controlling circRNA biogenesis?
- Are there still undiscovered functional aspects and mechanisms of circRNAs?
- How do circRNAs function in already well-characterized molecular pathways?
- To what extent would deeper understanding and utilization of circRNAs help improve human health?

## Introduction

Circular RNAs (circRNAs) are covalently closed circular RNA molecules recently reconsidered for their important roles in cancer and in other human diseases^1–3^. Since 2013, when Memczak et al.^4^ reported that circRNAs act as post-transcriptional regulators, additional circRNAs have been identified, implying an important regulatory potential for this class of molecules. It is currently broadly recognized that circRNAs have significant roles to play in cell proliferation^5–7^, motility and metastasis^5–9^, as well as in cell cycle progression^10^, angiogenesis^11,12^, and apoptosis^13^.

To exemplify the biomedical potential of circRNAs, we briefly review the biogenesis of circRNAs and then describe the role of some circRNAs in head and neck squamous cell carcinoma, breast and lung cancer. Among neurological disorders, we focus herein on Alzheimer disease. In addition, we report examples of circRNAs playing functional roles in cardiovascular diseases and in diabetes. Although the sponging mechanism of circRNAs toward microRNAs has emerged as the most common mechanism of action, additional modes of action have been proposed. CircRNAs can interact with proteins and some are translated into novel polypeptides or act as transcriptional regulators^2,14–21^. Presumably, as many circRNAs are being characterized, additional modes of action will be uncovered soon. CircRNAs are involved in many signaling pathways and some of these molecular pathways have been already characterized for their important roles in human diseases and they are subjects of clinical trials^22,23^. These characteristics, together with their presence in accessible body fluids, such as saliva, blood, and urine, make the circRNAs promising therapeutic targets and potential biomarkers for human diseases^24–26^.

## Biogenesis and function of circRNAs

CircRNAs can derive from exons, introns, antisense, 5′ or 3′ untranslated and intergenic genomic regions^27^. Exonic circRNAs (ecircRNAs) represent the most abundant species and they are produced by a “back-splicing” mechanism. During the biogenesis process, a downstream 5′ splice site of an exon is joined to an upstream 3′ splice site of the same or another exon, involving single or multiple exons^1,28–32^. The molecules derived from this mechanism form a closed circular transcript and an alternatively spliced linear RNA with skipped exons^31^. Thus, the mechanism that generates circRNAs uses the canonical spliceosomal machinery^31^. As a consequence, transcription of circRNAs competes with canonical pre-mRNA splicing and affects the rate of canonical gene expression^28^.

**Fig. 1:**
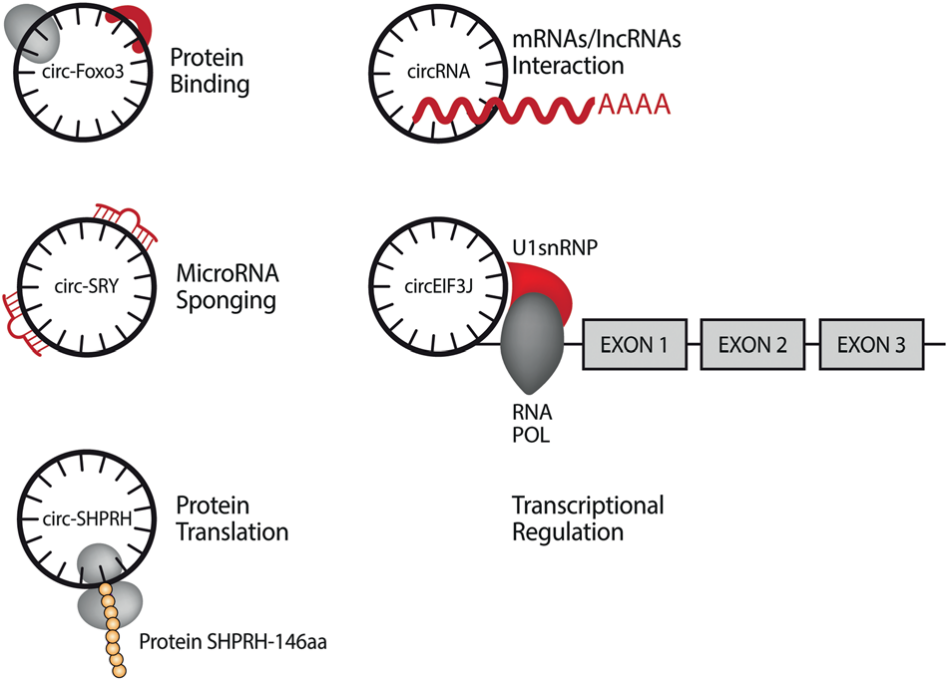
circRNA functions. CircRNAs can interact with proteins and other RNAs, acting as microRNA sponge, regulate the transcription and in some cases they can also be translated in proteins.

One of the best-described mechanisms explaining the biological function of circRNAs is the ability to effectively sponge microRNAs^33^ (Fig. 1). The circ-SRY^33^, CDR1as^4,33,34^, circ-ITCH^35^, circHIPK3 (ref. ^36^), circ_000984 (ref. ^37^), circ-TTBK2 (ref. ^38^), and circPVT1 (ref. ^39^) are examples of circRNAs that act as miRNA sponges (also called competing endogenous RNA; ceRNA). CircRNAs function as ceRNA via microRNA (miRNA) sequestration, by binding to miRNA response elements (MREs)^4^. Each circRNA can have many MREs on the miRNA target and the number of MREs is related to the length of the circRNAs themselves^4^.

CircRNAs can also interact with proteins (Fig. 1). Examples of circRNAs interacting with proteins include circ-PABPN^14^, circ-Foxo3 (ref. ^10^), and circ-Amotl1 (ref. ^15^). RNA-binding proteins (RBPs) specifically interact with RNA molecules to form ribonucleoprotein complexes^14^. The RBP HuR can bind circ-PABPN1 in human cervical carcinoma HeLa cells and it is responsible for the translation rate of the PABPN1 gene^14^ (Fig. 1). The RNA-binding protein quaking-5 (QKI-5) promotes circRNA biogenesis during epithelial-to-mesenchymal transition (EMT) through interaction with introns flanking the circRNA-forming exons^16^. Another protein that regulates circRNA biogenesis is the splicing factor called muscleblind (MBL). MBL promotes the formation of circMBL through interaction with introns flanking the circRNA itself^28^.

There are several examples of circRNA-protein interactions in the cancer context (Fig. 1). The tumor suppressor circ-Foxo3 interacts with CDK2 and p21 to form a ternary complex and inhibit cell cycle progression in cancer^10^. The oncogenic circRNA circ-Amotl1 promotes cell growth through an interaction with the proto-oncogene *c-MYC*. Circ-Amotl1 is able to increase the retention of nuclear c-MYC, promote c-myc stability, and up-regulating c-myc targets^15^.

There is also evidence showing that circRNAs can be translated into functional proteins (Fig. 1). Circ-ZNF609 is one of the first examples described of a circRNA that can be translated into a protein. Circ-ZNF609 is involved in the regulation of myoblast proliferation^17^.

The circular form of the SNF2 histone linker PHD RING helicase (SHPRH) gene, which encodes the protein SHPRH-146aa represents an additional example^18^. Both circ-SHPRH and SHPRH-146aa are highly expressed in normal human brains and their expression was found to be down-regulated in glioblastoma, suggesting a tumor suppressor role^18^. In a similar way, Zheng et al.^19^ identified circPPP1R12A, which is up-regulated in colon cancer (CC) and can be translated into a protein contributing to the rapid proliferation of CC cells via the Hippo-YAP pathway.

Finally, it is well accepted that intronic circRNAs (ciRNAs) and exon–intron circRNAs (ElciRNAs) can act as transcriptional regulators (Fig. 1)^20,21^. The intronic circRNA ci-ankrd52 is able to regulate its parental gene expression by modulating RNA polymerase II’s elongation activity^21^. Similarly, two ElciRNAs, circEIF3J and circPAIP2, are able to regulate the expression of their parental genes through a specific RNA–RNA interaction between U1 snRNA and the circRNA^20^. More recently Stoll et al.^40^ showed that the intronic circle ci-Ins2, located mainly in the nucleus of pancreatic β cells, is able to regulate insulin secretion through interaction with the TAR DNA-binding protein 43 kDa (TDP-43).

## CircRNAs in head and neck squamous cell carcinoma

Head and neck cancers represent the sixth most common cancer worldwide^41,42^. This cancer usually initiates in the squamous cells that line the mucosal surfaces inside the head and neck and can arise from the mucosal surfaces of the oral cavity (OSCC), oropharynx (OPSCC), and larynx. Head and neck cancers can also begin in the salivary glands and in paranasal sinuses and nasal cavities^41,42^. We showed that the circRNA circPVT1 acts as an oncogene in head and neck squamous cell carcinoma (HNSCC)^39^. CircPVT1 expression is regulated through the mut-p53/YAP/TEAD complex binding its own promoter, which is independent from the host gene PVT1 promoter^39^. CircPVT1 is overexpressed in tumors compared to matched-non-tumoral tissues and its expression is particularly high in patients with TP53 mutations^39^. This is an example of a circRNA acting as an oncogene and modulating the expression of miR-497-5p and some of its targets, such as aurka, mki67, and bub1, all genes involved in the control of cell proliferation. This is in line with the known role of miR-497-5p as a tumor suppressor in several cancers^39,43–46^.

Using high-throughput sequencing and RT-qPCR, Li et al.^47^ showed that hsa_circ_0008309 is down-regulated in OSCC tissues relative to paired adjacent normal tissues (ANTs)^47,48^ and statistically correlated with pathological differentiation^48^. Intriguingly, bioinformatics analysis showed that hsa_circ_0008309 might function within a molecular network involving miR-1290, miR-136-5p, miR-382-5p and the ATXN1 gene, coding for the DNA-binding protein Ataxin-1 (refs. ^47,48^).

Xuan et al.^8^ analyzed the circRNA expression in a cohort of Laryngeal squamous cell carcinoma (LSCC) tissues. They found that two circRNAs, hsa_circRNA_100855 and hsa_circRNA_104912, were respectively up- and down-regulated in cancer tissues in comparison to the corresponding adjacent non-neoplastic tissues^8^. Patients with T3-4 stage, neck nodal metastasis, or advanced clinical stage had higher hsa_circRNA_100855 expression and a lower hsa_circRNA_104912 expression^8,48^.

CircHIPK3 is highly expressed in nasopharyngeal carcinoma (NPC)^9^. The silencing of circHIPK3 can reduce cell proliferation, migration, and invasion in vitro and it can repress tumor growth and metastasis in vivo^9^. CircHIPK3 functions in NPC by sponging the miR-4288, which in turn targets the E74-like ETS transcription factor 3 (ELF3)^9^. Studying the circHIPK3-miR-4288-ELF3 molecular pathway could instruct ways to identify new therapeutic strategies focused on this regulatory loop.

## CircRNAs in breast cancer

Breast cancer is the most common cancer in females and it can be classified in three major cancer subtypes according to estrogen or progesterone receptor expression and *ERBB2* gene amplification: hormone receptor positive/ERBB2 negative (HR+/ERBB2-), ERBB2 positive (ERBB2+), and triple-negative^49^.

Galasso et al. performed a pilot study in which they described one of the first panels of circRNAs expressed in breast cancer by analyzing RNA sequencing data from five paired breast cancer samples^50^. At the same time, Nair et al.^51^ developed an automated workflow called Circ-Seq to identify circRNAs in breast tumors and breast cancer cell lines. A recent work identified 235 differentially expressed circRNAs in breast cancer through high-throughput circRNA microarray analysis^52^. Among all the modulated circRNAs, circTADA2A-E6 (hsa_circ_0006220) and circTADA2A-E5/E6 (hsa_circ_0043278) were ranked in the top five down-regulated circRNAs by microarray analysis^52^. In particular, circTADA2A-E6 sponges miR-203a-3p and functions as a tumor suppressor by inhibiting cell proliferation, migration, and metastasis. The *SOCS3* gene was predicted as a downstream target gene of the circTADA2A-E6/miR-203a-3p axis^52^, and a previous study reported that miR-203a-3p promotes cell proliferation by targeting SOCS3 in MCF-7 cells^53^. These results show that the circTADA2A-E6/miR-203a-3p/*SOCS3* axis plays an important role in the inhibition of breast cancer progression.

CircRNA-000911 is another circRNA acting in breast cancer as a tumor suppressor^54^. Wang et al.^54^ showed that circRNA-000911 suppresses the proliferative, migratory, and invasive capacities of breast cancer cells by sponging miR-449a and releasing Notch1, a functional target of miR-449a. This mechanism includes the involvement of Ago2, an essential protein for circRNA sponge activity^4,55^. The consequence of circRNA-000911 down-regulation in breast cancer is the up-regulation of miR-449a and down-regulation of Notch1. Importantly, one downstream effector of Notch1 is the nuclear factor-kB (kB), which normally promotes breast cancer tumorigenesis and progression^54^.

The circRNA circEPSTI1 (hsa_ circRNA_000479) is up-regulated in breast cancer and it is a prognostic marker and mediator of triple-negative breast cancer (TNBC) progression^56^. CircEPSTI1 behaves as an oncogene promoting TNBC cell proliferation in vitro and in vivo, and it is able to sponge both miR-4753 and miR-6809 (ref. ^56^). BCL11A is a direct target gene of both miRNAs, and it is inhibited as a consequence of circEPSTI1 knockdown. It follows that the circEPSTI1-miR-4753/6809-BCL11A axis could be an interesting pathway to investigate in order to identify new therapeutic strategies for the treatment of TNBC^56^.

CircANKS1B is another circRNA up-regulated in TNBC and its expression is associated with both lymph node metastasis and advanced clinical stage^57^. CircANKS1B is able to sponge miR-148a-3p and miR-152-3p, thereby increases the expression of transcription factor USF1, which in turn up-regulates TGF-β1 expression^57^. The up-regulation of TGF-β1 results in activation of the TGF-β1/Smad signaling pathway, promoting epithelial-to-mesenchymal transition (EMT)^57^. The results suggest that circANKS1B is an interesting circRNAs to study in order to find alternative therapeutic strategies for inhibiting breast cancer metastasis.

## CircRNAs in lung cancer

Lung cancer is one of the most common cancers in the world with 5-year survival rates varying from 92 to 0%, depending on disease stage at diagnosis^58^.

Circ-ITCH is generated from several exons of the ITCH E3 ubiquitin protein ligase (ITCH) and it shares the miR-7 and miR-214 binding sites with the three-prime untranslated regions (3′-UTR) of its parental gene ITCH^59^. Circ-ITCH plays an inhibitory role in lung cancer progression by sponging miR-7 and miR-214 and regulating the expression of ITCH^59,60^. ITCH negatively regulates canonical Wnt signaling by targeting the dishevelled-2 (Dvl2) protein^61^. In lung cancer the down-regulation of circ-ITCH brings to an increase of miR-7 and miR-214, thereby to a decrease of their target gene, ITCH. As a consequence, the Wnt/β-catenin pathway is enhanced, thereby promoting the development and progression of cancer^59,60^. Another circRNA that indirectly affects ITCH expression is hsa_circ_0043256. The circRNA hsa_circ_0043256 is able to sponge miR-1252, which binds the ITCH 3′-UTR^62^. Both circRNAs, circ-ITCH and hsa-circ_0043256, behave as tumor suppressors in lung cancer and their combined action could be used to design new strategies for the treatment of this malignancy.

In contrast to circ-ITCH, hsa_circ_0012673 is overexpressed in lung adenocarcinoma and promotes cell proliferation through the miR-22/ErbB3 pathway^63^. Hsa_circ_0012673 is able to sponge miR-22, which targets ERBB3/HER3, an important receptor tyrosine kinase in lung adenocarcinoma. ERBB3/HER3 is a member of the epidermal growth factor receptor (EGFR/ERBB) family^64^ and EGFR mutations were characterized for their important role in lung cancer^65^.

## CircRNA in Alzheimer’s disease

Alzheimer’s disease (AD) is the most prevalent cause of dementia affecting millions of people worldwide^66^. AD is a progressive and neurodegenerative disorder characterized by widespread neuronal atrophy and two histopathological hallmarks: extracellular senile plaques consisting of amyloid-β peptides, and intracellular neurofibrillary tangles composed of abnormally hyperphosphorylated Tau protein^66^.

Dube et al.^67^ generated RNA-seq data from individuals with and without AD to quantify cortical circRNA expression. The results showed that there are significant associations between circRNA expression and AD diagnosis, clinical dementia severity, and neuropathological severity^67^. Interestingly, circRNA expression changes can be observed early on, in pre-symptomatic AD and in autosomal dominant AD^67^. The microtubule-associated Tau protein plays a central role in AD since it is responsible for amyloid-beta induced neuronal cell death^68^. The MAPT gene generates the Tau protein. Using a PCR screen of RNA from human brain tissues, Welden et al. showed that the *MAPT* locus generates circRNAs through a backsplicing mechanism, but the role of these circRNAs is still unclear^69^.

Similarly, CDR1as has been one of the first circRNAs that were characterized. It derives from the cerebellar degeneration-related protein 1 antisense transcript (CDR1AS) and contains over 70 conventional binding sites for miR-7^4,33,70^. Down-regulation of CDR1as causes up-regulation of miR-7 and, consequently, negative regulation of its respective targets, such as ubiquitin protein ligase A (UBE2A)^71–73^. UBE2A is important for clearing amyloid peptides and it was found depleted in the AD brain^71–73^.

## CircRNAs in cardiovascular diseases

RNA-Seq analysis of ribosome-depleted libraries from hearts of human, mouse, and rats origins, detected more than 9000 candidate circRNAs for each species^74^. A similar analysis listed more than 15,000 cardiac circRNAs in humans^75^. Although the study showed no statistically significant circRNA that was differentially expressed in diseased hearts compared to healthy hearts, other studies are needed to elucidate the role of circRNAs in cardiac diseases^75^. On the other hand, the analysis found significant differential expressed circRNAs during cardiomyocyte differentiation^75^.

Many of the identified cardiac circRNAs are yet uncharacterized in terms of their specific function. Nevertheless, the identification of cardiac circRNAs represents a potential strategy to use circRNAs as target molecules in the prevention and treatment of cardiovascular diseases.

The first circRNA described with a cardioprotective role was the heart-related circRNA, HRCR. This circRNA acts as a miR-223 sponge to inhibit cardiac hypertrophy and heart failure^76^. MiR-223 is able to suppress the expression level of its target, ARC, the apoptosis repressor with CARD domain protein. HRCR acts as an anti-hypertrophic molecule due to its sponging mechanism toward miR-223, which causes up-regulation of ARC^76^.

More recently, circFndc3b was identified as another circRNA involved in cardioprotection. CircFndc3b interacts with the RNA-binding protein Fused in Sarcoma (FUS) to regulate VEGF expression and signaling^12^. Acting on the FUS/VEGF-A axis, circFBDc3b is able to enhance angiogenesis and retard cardiomyocytes and endothelial cell apoptosis^12^.

Yet another circRNA, Cdr1as (ciRS-7), acts as a miR-7a sponge in myocardial cells^77^. It was shown that ciRS-7 induces apoptosis in myocardial infarction (MI) in mice by means of increasing caspase-3 activity. CiRS-7 is up-regulated in infarcted hearts, and it is able to inhibit the miR-7a mediated cardiomyocyte protection against MI injury acting as a miRNA sponge^77,78^. CiRS-7’s sponge mechanism toward miR-7a determines the up-regulation of two miR-7a targets, PARP and SP1. These proteins play pro-apoptotic roles during MI^77^.

MFACR (mitochondrial fission and apoptosis-related circRNA) regulates mitochondrial fission and apoptosis in the heart, while acting as a miRNA sponge for miR-652-3p^79^. MiR-652-3p down-regulates its target, MTP18, a nuclear-encoded mitochondrial membrane protein that contributes to mitochondrial fission in mammalian cells^79,80^. As a result, the MFACR-activated pathway instigates cardiomyocyte death through miR-652-3p-dependent up-regulation of MTP18 expression^79^.

Another circRNA involved in cardiomyocyte apoptotic events is circNCX1, which is generated from the sodium/calcium exchanger 1 (ncx1) gene^81^. circNCX1 acts as a miRNA sponge for miR-133a-3p, which is able to target the pro-apoptotic gene called Cell Death-Inducing p53-target Protein 1 (CDIP1). Importantly, miR-133a-3p plays a cardioprotective role and it is suppressed by the circNCX1 sponge mechanism^81^. This is an example of circRNAs that enhances damage following a MI event, primarily by promoting apoptosis of cardiomyocytes^81^.

## CircRNAs in diabetes

Diabetes is a group of metabolic disorders all characterized by hyperglycemia, namely high levels of sugar in the blood. This condition is associated with various pathological states, such as cardiovascular disease, retinopathy, nephropathy, and neuropathy^82^.

A recent work showed the human pancreatic islets express thousands of circRNAs^83^. The circRNA Cdr1as is already known for its miR-7 sponging activity in embryonic zebrafish brains and in infarcted hearts^4,34,84^. Moreover, Cdr1as is able to affect miR-7 function in adult islet cells^84^. Xu et al.^84^ showed that miR-7 is highly expressed in islet cells, and its overexpression in transgenic mice β-cells causes diabetes due to impaired insulin secretion and β cell dedifferentiation. Cdr1as promotes insulin secretion by sponging miR-7 in islet cells^84^. Hence, the interaction between Cdr1as and miR-7 in insulin secretion may become a new therapeutic target for improving β cell function in diabetes^84^.

The circRNA circHIPK3 was found up-regulated in retinas and retinal endothelial cells of patients with diabetes. CircHIPK3 is able to regulate the retinal vascular endothelial function while sponging miR-30a-3p^85^. As a consequence of its action as miRNA sponge, circHIPK3 increases the expression of VEGFC, FZD4, and WNT2, leading to endothelial proliferation and vascular dysfunction^85^. It follows that circHIPK3 could serve as a valid target for diabetic retinopathy.

## CircRNAs as potential disease biomarkers

Both prognostic biomarkers and markers that predict responses to a drug or other treatment modalities must bear high specificity for a given pathophysiological condition and a highly reproducible detection capacity. Accordingly, the renewed identification of circRNAs has opened a new potential strategy for diagnosis and for monitoring progression of different human diseases (Table 1). This is primarily due to the high tissue specificity of circRNAs, their relatively high stability in tissues and body fluids, as well as ease of detection using rather simple technologies, such as real-time PCR^1,86,87^. CircRNAs are highly abundant in blood^25^ and there are also evidences of circRNAs in urine samples^26^, for example to assist monitoring of patients who have undergone kidney transplantation, or for diagnosis of patients with bladder cancer^26,88^.

**Table 1:** CircRNAs as potential biomarkers of human diseases.

CircRNA	Disease/expression	Detection method	Refs.
hsa_circ_0000190	GC/decreased	qRT-PCR	^96^
hsa_circ_002059	GC/decreased	qRT-PCR	^97^
circFARSA	NSCLC/increased	RNA-seq; qRT-PCR	^98^
F-circEA-2a	NSCLC/increased	qRT-PCR	^99^
hsa_circ_0027089	LIHC/Increased	Microarray; qRT-PCR	^100^
circ-LPAR1	AD/increased	Microarray; qRT-PCR	^102^
circ-AXL	AD/increased	Microarray; qRT-PCR	^102^
circ-GPHN	AD/increased	Microarray; qRT-PCR	^102^
circ-PCCA	AD/decreased	Microarray; qRT-PCR	^102^
circ-HAUS4	AD/decreased	Microarray; qRT-PCR	^102^
circ-KIF18B	AD/decreased	Microarray; qRT-PCR	^102^
circ-TTC39C	AD/decreased	Microarray; qRT-PCR	^102^
hsa_circRNA_405619	AD/increased	Microarray; qRT-PCR	^103^
hsa_circRNA_000843	AD/increased	Microarray; qRT-PCR	^103^
hsa_circRNA_100861	AD/decreased	Microarray; qRT-PCR	^103^
hsa_circRNA_102448	AD/decreased	Microarray; qRT-PCR	^103^
hsa_circRNA_025016	*PAF/*increased	Microarray; qRT-PCR	^106,107^
MICRA	LVD/increased	qRT-PCR	^106,107^
circANRIL	ATH/Increased	qRT-PCR	^108^

Listed are examples of circRNAs that might serve as biomarkers of various diseases.

*GC* gastric cancer, *NSCLC* non-small cell lung cancer, *LIHC* liver hepatocellular carcinoma, *AD* Alzheimer’s disease, *PAF* postoperative atrial fibrillation, *LVD*: left ventricular dysfunction, *ATH* atherosclerosis.

Importantly, several recent studies reported the presence of circRNAs in extracellular vesicles, mainly exosomes, which are targets for discovery of additional types of new biomarkers^89–91^. The abundance and diversity of circRNAs in human blood exosomes is already available in a database called exoRBase^92^. Likewise, circRNAs with diagnostic potential have been found in urine exosomes^93,94^. Additionally, another database, MiOncoCirc, was created based on sequencing of more than 2000 tumor samples, and many urine circRNAs were identified as possible biomarkers for prostate cancer^95^.

There are several papers that have shown correlations between expression of specific circRNAs and tumor grade, size, metastatic spread, and lymph node involvement. This is the case of hsa_circ_002059 and hsa_circ_0000190, which were found to be decreased in plasma of patients with gastric cancer^96,97^. Likewise, it has been reported that circFARSA is elevated in plasma of patients with non-small-cell lung cancer (NSCLC), in direct association with tumor cell aggressiveness in vitro^98^. The circRNA F-circEA-2a is another candidate biomarker in NSCLC. Generated from the prevalent fusion gene in lung cancer, *EML4-ALK*, circRNA F-circEA-2a appears elevated in plasma samples^99^.

A screening seeking differentially expressed circRNAs in plasma of patients with hepatocellular carcinoma related to the hepatitis B virus, reported elevated expression of hsa_circ_0027089 and classified it as a potential biomarker^100^. Additionally, an atlas of Blood-Based Biomarkers for Early Diagnosis of Cancers (BBcancer) has recently been established^101^. It includes data from 5000 samples across 15 different types of cancer^101^.

A recent study evaluated the presence of circRNAs in cerebrospinal fluid of patients with Alzheimer’s disease (AD) and found 112 up-regulated and 51 down-regulated circRNAs^102^. Some of these circRNAs were confirmed by real-time PCR, with circ-LPAR1, circ-AXL, and circ-GPHN elevated and circ-PCCA, circ-HAUS4, circ-KIF18B, and circ-TTC39C decreased in patients with AD^102^.

Another study showed that it is possible to differentiate patients with AD and healthy individuals by testing the expression of circRNAs in peripheral blood mononuclear cells (PMBCs)^103^. Hsa_circRNA_405619 and hsa_circRNA_000843 were shown to be elevated in PBMCs of patients with AD in comparison to healthy individuals, while hsa_circRNA_100861 and hsa_circRNA_102448 appear decreased in the same patients^103^.

Several published reports relate to circRNAs in different cardiovascular diseases^104,105^. The presence of hsa_circRNA_025016 in the plasma of patients is able to predict postoperative atrial fibrillation, while MICRA (myocardial infarction-associated circRNA) can help predicting left ventricular dysfunction in patients with acute myocardial infarction^106,107^. Similarly, in addition to being much more expressed than its linear form, an isoform of circANRIL has been shown to be elevated in whole blood of cardiac patients and playing an atheroprotective role, unlike its linear counterpart, which appears to play a proatherogenic role^108^.

## CircRNAs implicated in molecular pathways disclose their potential use as therapeutic molecules

**Fig. 2:**
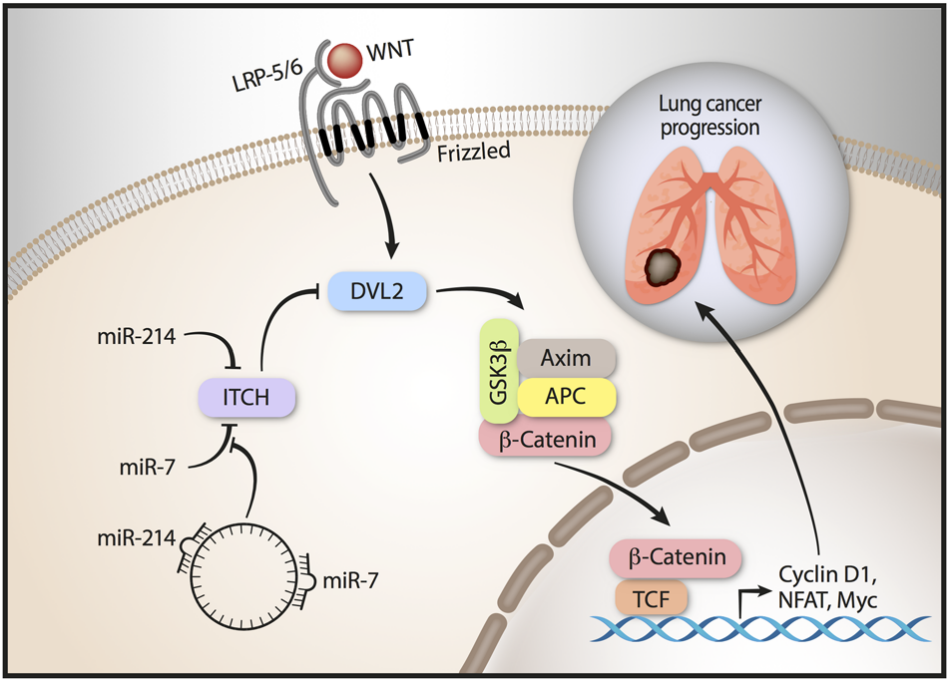
circ-ITCH working in the WNT signaling pathway. Circ-ITCH negatively regulates the canonical Wnt signaling in lung cancer by sponging miR-7 and miR-214 and regulating the expression of ITCH.

Although several circRNAs have been found to be either up- or down-regulated in human tissues, not always their specific role in molecular pathways has been elucidated. Specific circRNAs act in the Wnt signal transduction pathway: circRNA ITCH is active in lung cancer^59^ and cZNF292 is active in glioma^109^ (Fig. 2). Silencing cZNF292 blocked glioma cell cycle progression by means of inhibiting the Wnt/ß-catenin signaling pathway^109^.

Wnt signaling can be divided into β-catenin-dependent (or canonical) and β-catenin-independent (or non-canonical) signaling^22,110,111^. This pathway plays a critical role during embryonic development, including cell fate specification, cell proliferation, and cell migration. Moreover, the role of Wnt signaling has been well characterized in several diseases, such as cancer, diabetes, and cardiovascular disorders^112–114^. Accordingly, clinical trials that tested Wnt signaling drugs have shown promising outcomes, and circRNAs affecting the Wnt pathway might serve as targets for new therapies^22,115^.

**Fig. 3:**
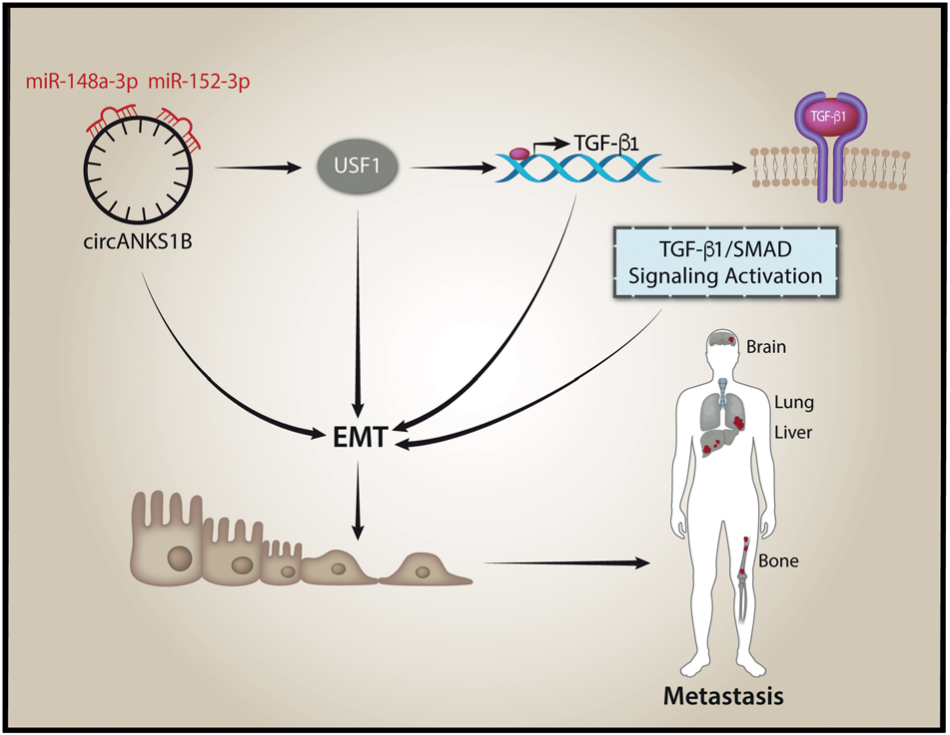
circANKS1B working in the TGF- β1 signaling pathway. CircANKS1B promotes the epithelial-to-mesenchymal transition (EMT) in triple-negative breast cancer sponging the miR-148a-3p and miR-152-3p, enhancing the expression of transcription factor USF1 and activating the TGF-β1/Smad signaling pathway.

CircANKS1B promotes the epithelial-to-mesenchymal transition (EMT) in triple-negative breast cancer (TNBC)^57^ (Fig. 3). EMT takes place in a diverse range of physiological and pathological conditions^116^. The molecular reprogramming occurring during EMT is orchestrated by a complex combination of factors, possibly including circRNAs. The biogenesis of numerous circRNAs is promoted during EMT transition by the RNA-binding protein quaking-5 (QKI-5)^16^. Recently, several clinical trials have been launched based on the current knowledge of EMT heterogeneity and plasticity^117^. The next challenge will be to include circRNAs as biomarkers or pharmacological targets in the protocols of new clinical trials addressing EMT.

**Fig. 4:**
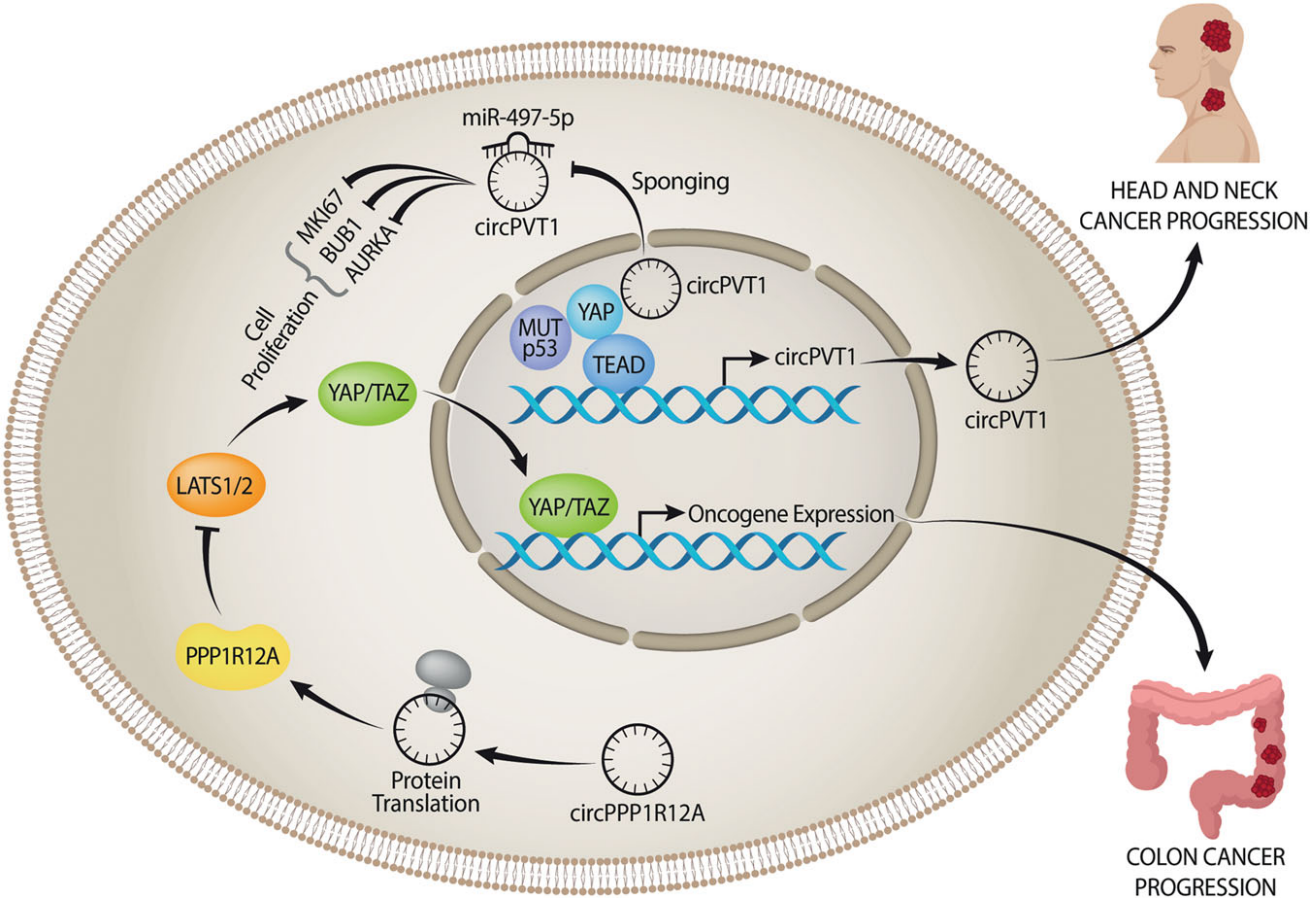
circPVT1 and circPPP1R12A working in the Hippo signaling pathway. The circPVT1 acts as oncogene in head and neck squamous cell carcinoma sponging the miR-497-5p and binding the complex YAP/mutp53. The circPPP1R12A can be translated into a protein and acts as oncogene in colon cancer where it is able to activated the proliferation of the tumor cells via theHippo-YAP pathway.

The circPVT1 and the circPPP1R12A act within the Hippo-YAP signaling pathway, respectively in head and neck squamous cell carcinoma and in colon cancer^19,39^ (Fig. 4). The Hippo pathway is recognized as an evolutionarily conserved signal transduction pathway that controls proliferation, organ size, and shape during development^23^. Moreover, the Hippo pathway is involved in multiple physiological processes, such as tissue growth, regeneration, and repair, maintaining the tissue homeostasis^23^. Hippo signaling plays an important role as a tumor suppressor in cancer and its deregulation is a key feature for cancer development, progression, and resistance to cancer treatment^23,118,119^. We showed that the mutant form of p53 (mut-p53) physically interacts with the transcriptional cofactor Yes-Associated Protein (YAP) in breast cancer^120^. YAP and TAZ are the main effectors of the Hippo pathway^120^. Hippo pathway inactivation determines the translocation to the nucleus of YAP and TAZ that regulate transcriptional activation in collaboration with mut-p53. In this context, Hippo effectors YAP and TAZ can act either as tumor suppressors, when located in the cytoplasm, or as oncogenes in the nucleus. In its wild type conformation, p53 works as a tumor suppressor regulating the cellular homeostasis^23^. At the same time the “p53 status”, wild type or mutant, can be considered a critical point in determining the tumor suppressor or oncogenic activity of the Hippo pathway^23^. Currently, there are several pathway modulators of the Hippo pathway that are subject of clinical development, such as the Verterpofin who inhibits the YAP–TEAD interaction^121^ or the PRIMA1-MET that restores the pro-apoptotic function of p53 with consequent activation of downstream target genes^122^. Importantly, we showed that YAP binds circPVT1 in head and neck squamous cell carcinoma. Moreover, we demonstrated that mut-p53 stabilizes the YAP/circPVT1 complex^39^. Thus, the current knowledge of circRNAs and their interaction with the Hippo pathway are expected to open new ways for the development of novel and more effective drugs.

## Divergent views of circRNA biogenesis and their mode of action

Despite the great interest that the circRNAs are raising in the scientific community, there are still some important questions regarding their biogenesis and function. The presence of repetitive inverted Alu elements flanking exons favors RNA circularization^123^. However, Zhang et al. demonstrated another mechanism not dependent on repetitive sequences for the generation of circRNAs, and occurring by the pairing between complementary sequences in introns flanking exons^32^. Another regulatory mechanism for circRNA biogenesis uses the ADAR protein, which is capable of modifying nucleotides in intronic repeat sequences^29^. Ivanov et al.^29^ demonstrated that ADAR antagonizes the expression of several circRNAs by editing intronic sites that flank exons and promote back-splicing. It is becoming clearer that introns are more important sequences for circRNAs biogenesis than initially anticipated, and that specific proteins might regulate backsplicing. Apparently, several different mechanisms control circRNA biogenesis, but it is yet unclear how do they work and which one, if any, is predominant over the others.

The unique circular configuration confers to cirRNAs not only resistance to digestion by ribonucleases, but it also translates to a longer half-life compared to the respective mRNAs^124–126^. As a consequence, circRNA levels are typically reduced in rapidly proliferating cells, such as in cancer cells. Thus, the association between lower circRNA levels and cancer could be due, in some cases, to a simple dilution effect mediated by cell division, as in colorectal cancer^127^. Hence, the meaning of circRNA expression levels should be carefully evaluated based on the specific cellular context. In addition, many circRNAs are sensitive to RNase R treatments, thus contradicting claims related to high stability of these molecules^128^.

Although the microRNA sponging mechanism is the better-described role for circRNAs, many circRNAs putatively act as sponges toward only a single, or very few miRNA targets^129^. Notably, there are some prerequisites that should be fulfilled in order to identify a circRNA as a putative ceRNA: the presence of multiple binding sites for the miRNA target, relatively high abundance of the circRNA, a miRNA target with fewer target genes, and a circRNA with a better affinity toward the miRNA than the mRNAs–miRNA affinity^130^. Moreover, a circRNA that triggers the degradation process of a target miRNA, and not only inhibits the interaction between miRNA and mRNA, might act as a better ceRNA candidate^129^.

The most common approach for assessing the sponging mechanism is the ectopic overexpression of binding sites for a specific miRNA. However, the result of this kind of experiment should be interpreted with caution since it could be biased by the introduction of sufficiently high numbers of binding sites able to inhibit the activity of the miRNA in question^126^. At the same time, one should also keep in mind that if a circRNA only binds with the cognate miRNA and inhibits its function without degrading it, the abundance of the miRNA would not be affected. Piwecka et al.^131^ have shown that the choice between either degradation or functional inhibition depends on whether the binding sites connecting circRNAs and miRNAs are completely complementary or they only partially match each other. It follows that a reliable in silico analysis of putative binding sites for circRNAs on miRNA targets is an essential requirement. While most studies have shown repression of miRNAs, Hansen et al.^132^ showed that the interaction between Cdr1 and miR-671 would actually lead to degradation of this circRNA through AGO2 rather than by the expected miRNA degradation mode^132^. Therefore, it is possible that additional circRNA–miRNA interactions regulate RNA circles.

With a few exceptions, the majority of circRNAs are expressed at low levels in both normal and cancer cells; hence they are unlikely to have only secondary roles in cellular physiology. However, the cascade of events that a single circRNA might unleash can potentially be of great importance from the clinical point of view, as we have shown for the circRNA circPVT1 (ref. ^39^). The roles of circRNAs must be carefully assessed in light of the various processes of their biogenesis and degradation, in addition to their broad capabilities for interacting with miRNAs and proteins.

## Conclusions

The biochemical and molecular characteristics of circRNAs hold the promise that specific circles of RNA will be utilized in the future as disease biomarkers and pharmacological targets, thus opening new possibilities for early detection and treatment^133–135^. The large spectrum of mechanisms of action used by circRNAs makes the understanding of their role not only challenging but also promising in terms of resolving the complex molecular mechanisms activated in human disorders. Indeed, circRNAs can act as tumor suppressors or as oncogenes in oncology^136,137^. Likewise, circRNAs are involved in cardioprotection against heart failure, as well as mediate cardiomyocyte death in myocardial infarction^12,76–79^. Moreover, circRNAs are extensively expressed in the mammalian brain^138,139^. Networks of circRNAs, RNA-binding proteins and microRNAs play important roles in different human diseases, which reflects the complex regulatory potential of circRNAs. Hence, it is likely that the next few years will witness the discovery of more circRNAs and new modes of their action in human disorders.
